# Amplicon Sequencing of Variable 16S rRNA from Bacteria and ITS2 Regions from Fungi and Plants, Reveals Honeybee Susceptibility to Diseases Results from Their Forage Availability under Anthropogenic Landscapes

**DOI:** 10.3390/pathogens10030381

**Published:** 2021-03-22

**Authors:** Aneta A. Ptaszyńska, Przemyslaw Latoch, Paul J. Hurd, Andrew Polaszek, Joanna Michalska-Madej, Łukasz Grochowalski, Dominik Strapagiel, Sebastian Gnat, Daniel Załuski, Marek Gancarz, Robert Rusinek, Patcharin Krutmuang, Raquel Martín Hernández, Mariano Higes Pascual, Agata L. Starosta

**Affiliations:** 1 Department of Immunobiology, Faculty of Biology and Biotechnology, Institute of Biological Sciences, Maria Curie-Skłodowska University, Akademicka 19 Str., 20-033 Lublin, Poland; 2 School of Biological and Chemical Sciences, Queen Mary University of London, London E1 4NS, UK; p.j.hurd@qmul.ac.uk; 3 Polish-Japanese Academy of Information Technology, Koszykowa 86 Str., 02-008 Warsaw, Poland; przemyslawlatoch@gmail.com; 4 Laboratory of Gene Expression, ECOTECH-Complex, Maria Curie-Sklodowska University, ul. Gleboka 39, 20-612 Lublin, Poland; agata.starosta@umcs.pl; 5 Department of Life Sciences, Insects Division, Natural History Museum, London SW7 5BD, UK; a.polaszek@nhm.ac.uk; 6 Biobank Lab, Department of Molecular Biophysics, Faculty of Biology and Environmental Protection, University of Łódź, Pilarskiego 14/16, 90-231 Łódź, Poland; joanna.michalska@biol.uni.lodz.pl (J.M.-M.); lukasz.grochowalski@biol.uni.lodz.pl (Ł.G.); dominik.strapagiel@biol.uni.lodz.pl (D.S.); 7 Department of Veterinary Microbiology, Faculty of Veterinary Medicine, Institute of Preclinical Veterinary Sciences, University of Life Sciences, Akademicka 12, 20-033 Lublin, Poland; sebastian.gnat@up.lublin.pl; 8 Department of Pharmaceutical Botany and Pharmacognosy, Ludwik Rydygier Collegium Medicum, Nicolaus Copernicus University, Marie Curie-Skłodowska 9, 85-094 Bydgoszcz, Poland; daniel_zaluski@onet.eu; 9 Institute of Agrophysics, Polish Academy of Sciences, Doświadczalna 4 Str., 20-290 Lublin, Poland; m.gancarz@ipan.lublin.pl (M.G.); r.rusinek@ipan.lublin.pl (R.R.); 10 Faculty of Production and Power Engineering, University of Agriculture in Kraków, Balicka 116B, 30-149 Kraków, Poland; 11 Department of Entomology and Plant Pathology, Faculty of Agriculture, Chiang Mai University, Chiang Mai 50200, Thailand; patcharink26@gmail.com; 12 Research Center of Microbial Diversity and Sustainable Utilization, Faculty of Science, Chiang Mai University, Chiang Mai 50200, Thailand; 13 Centro de Investigación Apícola y Agroambiental (CIAPA), Laboratorio de Patología Apícola, IRIAF Instituto Regional de Investigación y Desarrollo Agroalimentario y Forestal, Consejería de Agricultura de la Junta de Comunidades de Castilla-La Mancha, Camino de San Martín s/n, 19180 Marchamalo, Spain; rmhernandez@jccm.es (R.M.H.); mhiges@jccm.es (M.H.P.); 14 Instituto de Recursos Humanos para la Ciencia y la Tecnología (INCRECYT-FEDER), Fundación Parque Científico y Tecnológico de Castilla—La Mancha, 02006 Albacete, Spain; 15 Department of Molecular Biology, Institute of Biological Sciences, Maria Curie-Sklodowska University, Akademicka 19 Str., 20-033 Lublin, Poland

**Keywords:** *Apis mellifera*, 16S rRNA, ITR2, NGS, *Nosema apis*, *Nosema ceranae*, *Nosema bombi*, *Acarapis woodi*, Trypanosomatida, *Crithidia* spp., neogregarines, *Apicystis* spp., antropocene, insectageddon, urban area, urban environment, bee biology

## Abstract

European *Apis mellifera* and Asian *Apis cerana* honeybees are essential crop pollinators. Microbiome studies can provide complex information on health and fitness of these insects in relation to environmental changes, and plant availability. Amplicon sequencing of variable regions of the 16S rRNA from bacteria and the internally transcribed spacer (ITS) regions from fungi and plants allow identification of the metabiome. These methods provide a tool for monitoring otherwise uncultured microbes isolated from the gut of the honeybees. They also help monitor the composition of the gut fungi and, intriguingly, pollen collected by the insect. Here, we present data from amplicon sequencing of the 16S rRNA from bacteria and ITS2 regions from fungi and plants derived from honeybees collected at various time points from anthropogenic landscapes such as urban areas in Poland, UK, Spain, Greece, and Thailand. We have analysed microbial content of honeybee intestine as well as fungi and pollens. Furthermore, isolated DNA was used as the template for screening pathogens: *Nosema apis*, *N. ceranae*, *N. bombi*, tracheal mite (*Acarapis woodi*), any organism in the parasitic order Trypanosomatida, including Crithidia spp. (i.e., *Crithidia mellificae*), neogregarines including *Mattesia* and *Apicystis* spp. (i.e., *Apicistis bombi*). We conclude that differences between samples were mainly influenced by the bacteria, plant pollen and fungi, respectively. Moreover, honeybees feeding on a sugar based diet were more prone to fungal pathogens (*Nosema ceranae*) and neogregarines. In most samples *Nosema* sp. and neogregarines parasitized the host bee at the same time. A higher load of fungi, and bacteria groups such as Firmicutes (*Lactobacillus*); ***γ***-proteobacteria, Neisseriaceae, and other unidentified bacteria was observed for *Nosema ceranae* and neogregarine infected honeybees. Healthy honeybees had a higher load of plant pollen, and bacteria groups such as: *Orbales*, *Gilliamella*, *Snodgrassella*, and Enterobacteriaceae. Finally, the period when honeybees switch to the winter generation (longer-lived forager honeybees) is the most sensitive to diet perturbations, and hence pathogen attack, for the whole beekeeping season. It is possible that evolutionary adaptation of bees fails to benefit them in the modern anthropomorphised environment.

## 1. Introduction

Next-generation sequencing (NGS) is a culture-independent method often used for studying entire microbial communities, and helps to understand how microbes influence health and diseases of humans and animals including the honeybee [1,2,3]. Adult honeybees harbour a specialized gut microbiota of relatively low complexity with diet as a major factor influencing differences in bacterial loads [4]. Although honeybee microbiome core species composition is quite consistent regardless of environmental, geographical and genetic differences between specimens [2], some studies indicate that it can be sensitive to infection, changes in diet, malnutrition and many anthropogenic activities, such as extensive pesticide use and urban land-use changes [5,6,7].

Species within the *Apis* genus share fewer than 10 core species members, including *Lactobacillus*, *Bifidobacterium*, *Neisseria*, *Pasteurella*, *Gluconobacter*, *Snodgrassella* and *Gilliamella* [8,9,10,11]. Bacteria present in the honeybee gut provide numerous beneficial effects: they help digest and absorb necessary compounds and microelements, protect against mild poisonings with xenobiotics, acidify their environment which protects the gut from pathogenic microbes, e.g., *Paenibacillus larvae* that causes foulbrood, or *N. ceranae* that causes nosemosis [12,13]. They also have immunomodulation effects improving bees’ immunity, strengthening the condition of the colony and prolonging bees’ lives [11]. From the kingdom Fungi, yeasts are prevalent organisms in every environment in which bees conduct their life cycle, and can be isolated, for example, from honey and nectar. Honey microflora is composed of Gram-positive bacteria and yeasts, such as *Saccharomyces rouxi, S. mellis*, *S. bisporus*, *S. roesi*, *S. bailli*, *S. heterogenicus*, *Pichia (Hansenula) anomala*. The pollen reserves flora, which is dominated by bacteria from the genera *Pseudomonas* and *Lactobacillus*, and fungi from the genera Saccharomyces, Candida and Cryptococcus, far outnumbers the microflora of the honey [14,15,16,17,18]. Surprisingly, the gut flora of healthy, free-flying bees contains only a few yeasts if any, and diseases, malnutrition, antibiotics and insecticides cause an increase in the number of yeasts [16,19,20]. Therefore, an increased number of yeast colonies isolated from bees’ guts may be considered as a stress indicator. However, recent work of Tauber et al. [5,21] suggested that, in general, yeasts are important during a younger bee’s life, which includes in-house duties to feed the hive, and that the yeast community becomes less essential to the honeybee after foraging begins. Honeybee colonies are complex super-organisms where social immune defences, natural homeostatic mechanisms, microbiome diversity and function play a major role in disease resistance. However, there is still little known about connections between, and variation among bee pathogens, bee microbiota and anthropogenic changes of environment.

Currently, in developed countries, anthropogenic landscapes are the most impacting features and include those created either directly by human activity, or indirectly by natural processes triggered by human activity [22,23,24]. Not only has human activity influenced geological features, but it has also considerably affected flora and fauna [25]. The loss in biodiversity is often described as the sixth mass extinction, and a slump in insect mass and biodiversity is so spectacular that the term Insectaggedon has been used to describe the phenomenon [26,27,28,29,30].

Recent research shows insects to be dying out eight times faster than mammals, birds, or reptiles [26,31,32]. Most noteworthy factors behind the decline of insects are inappropriate application of pesticides, increased use of fertilizers and intense agronomic activities, highly intensive farming, insect malnutrition caused by farmland monocultures, parasites, long-term drought, long-term lack of sun, especially accompanied by low temperatures, as well as viral, bacterial, and fungal diseases [24,33]. Currently special concern is being paid to the decline of pollinators, largely because of their essential ecosystem services [20]. Therefore, both for educational purposes and as a way to preserve pollinator populations many urban pollinator initiatives have arisen recently (e.g.,“Life + Urbanbees” [34], “City Bees” [35], “Urban Beekeeping” [36]). Urban colonies were shown to be more productive than rural ones, as they had access to a greater number and variety of plant species, allowing honeybees to diversify nectar sources and produce honey at a higher rate [37,38]. On the other hand, wild pollinators such as *Bombus* and *Lasioglossum* spp. were negatively affected by urbanization, but increasing the abundance and richness of floral resources could partially compensate this effect [39]. Effects of urbanization on bees are complex, variable and not well-understood [40,41]. Therefore, the aim of this study was to use the amplicon sequencing of variable 16S rRNA and ITS2 to screen honeybee colonies originating from different urban areas, and to check if “the bees really love the city”.

## 2. Results and Discussion

**Figure 1:**
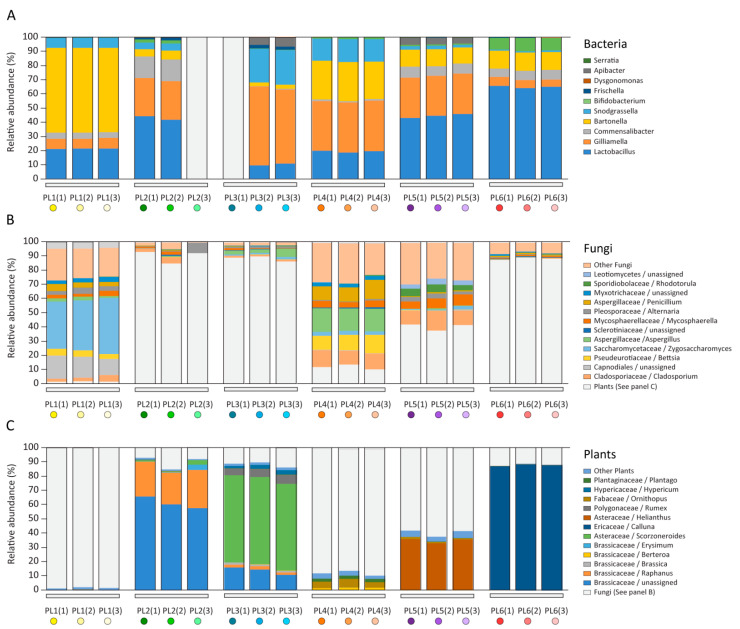
Composition of bacteria (**A**), fungi (**B**) and pollen (**C**) from Polish honeybee samples.

**Figure 2:**
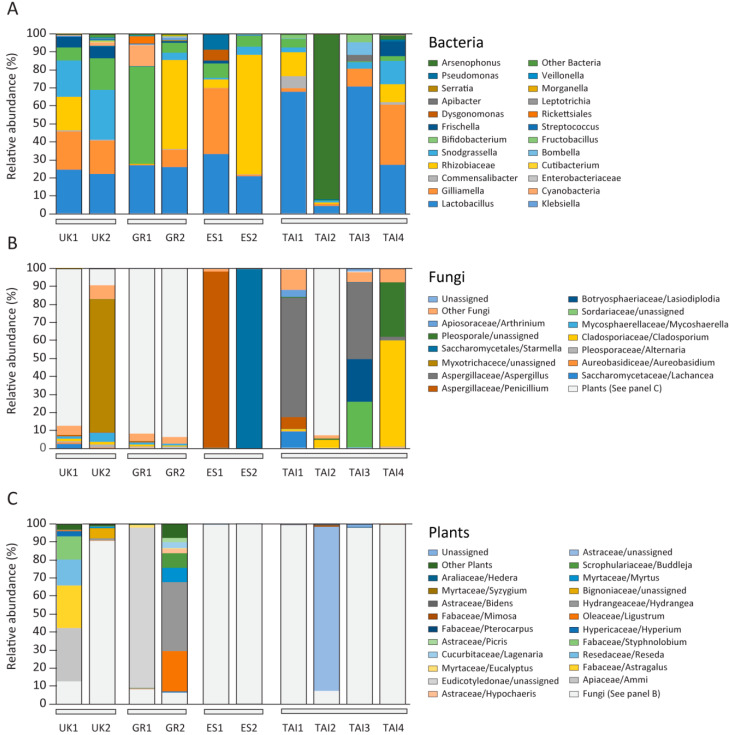
Composition of bacteria (**A**), fungi (**B**) and pollen (**C**) from UK, Greek, Spanish, and Thai bee samples.

Adult honeybees harbour specialized gut microbiota of relatively low complexity with five core bacterial strains [3,4,9,42,43]: *Lactobacillus* Firm-4 and Firm-5 (Firmicutes), *Giliamella* (γ-proteobacteria, Orbales), *Snodgrassella* (γ-proteobacteria, Neisseriaceae), *Bifidobacterium* (Actinobacteria) and a number of elective bacterial strains, including *Frischella* (γ-proteobacteria, Orbales), *Bartonella* (α-proteobacteria, Rhizobiales), *Commensalibacter* (α-proteobacteria, Acetobacterales) and *Bombella* (α-proteobacteria, Acetobacterales), which was also confirmed in this study (Figure 1 and Figure 2, Supplementary Materials Tables S1 and S2).

NGS was used successfully for taxonomic assessment of pollen and plants from many ecological and palynological studies, and to determine plant–pollinator interactions, or to confirm the floral composition of honey [3,44,45,46,47].

### 2.1. Microbiome and Pollen Composition of Honeybees from Poland (Differences over the Vegetation Season)

Following NGS analysis, it was possible to assign samples to the time that they were collected by comparing them with vegetation periods of nectar- and pollen-rich plants. From one location, 3 specimens (forager honeybees) were taken, as the representative and consistent number for each group (data adequacy confirmed by the principal component analysis (PCA) analysis of amplicons of the 16S rRNA from bacteria and the ITSs region from fungi and plants, Supplementary Materials Figure S1). Microbiome down to genera analysis enabled division of Polish honeybee samples into 6 sub-groups: PL1, PL2, PL3, PL4, PL5, and PL6 (Supplementary Materials Table S1).

PL1 forager honeybees were collected in April and 16 taxa were identified in total from 16S amplicon analysed and 164 taxa in ITS2 analyses. In PL1 a low number of pollen types was observed (1.81%, SD = 0.512 belonging primarily to the *Betula* sp. and *Urtica* sp. genera) and a high number of fungi (93.83%, SD = 0.554). The low number of pollen types may relate to the low availability of flowering resources at the start of the flowering season, when the bees have to consume the stored feeding solution in the colony, or after feeding by the beekeeper later in the season. A sugar-based diet can lead to higher yeast numbers as observed in PL1 (35.92%, SD = 3.208) (Supplementary Materials Tables S1 and S2). Furthermore, the pathogenic fungus *Nosema ceranae* was detected in the PL1 (Supplementary Materials Table S3).

PL2 forager honeybees were collected in May and 16 taxa were identified in total from 16S amplicons analysed and 147 taxa in ITS2. *Lactobacillus* spp. were the dominant bacteria in PL2 (43.02%, SD = 1.704) (Supplementary Materials Table S1). At the same time, the percentage of fungi was moderate (9.59%; SD = 4.476) with the prevalence of *Cladosporium* and a small content of fungi from other genera (Supplementary Materials Table S2). The dominant pollen content in the PL2 with 87.54% (SD = 3.886) came from plants from the *Brassicaceae*, mainly unassigned, and speciesof *Raphanus* (Supplementary Materials Table S1B) known for its high protein pollen content [31]. No pathogens were detected in PL2 (Supplementary Materials Table S3).

PL3 forager honeybees were collected in June and 18 taxa were identified in total from 16S amplicons analysed and 177 taxa in ITS2. This group shows a higher number of *γ-*proteobacteria from *Gilliamella* and *Snodgrassella* (54.03%, SD = 2.333) (Supplementary Materials Table S1). PL3 honeybees collected pollen from Polygonaceae, which contain moderate amounts of amino acids [48]. In this group, fungal load was moderate (11.25%, SD = 1.847) with *Aspergillus*, *Cladosporium* and *Mycosphaerella* (mainly transferred as bioaerosols by wind in the air) at the level of 3.76% (SD = 1.9486), 1.21% (SD = 0.0961), and 0.81% (SD = 0.624), respectively (Supplementary Materials Table S1B). No pathogens were detected in PL3 (Supplementary Materials Table S3).

PL4 forager honeybees were collected in July and 16 taxa were identified in total from 16S amplicons analysed and 322 taxa in ITS2. This group was differentiated on the basis of the highest fungal DNA loads (87.31%, SD = 1.680) and trace amounts of plant DNA (Supplementary Materials Table S1B). The fungi were mainly *Aspergillus* (15.98%, SD = 0.503), *Cladosporium* (10.91%, SD = 1.048), *Penicillum* (11.07%, SD = 2.190), and *Betsia* (11.20%, SD = 1.572) spp. The spores of the three first genera commonly appeared in the air (transferred as bioaerosols), but the presence of *Betsia* species is suggestive of poor health of the honeybee colony. The pollen mould (*B. alvei*) is a saprophyte that lives on the pollen stored combs, especially in temperate regions [49]. Furthermore, pathogens belonging to *N. ceranae* and neogregarines were detected in the PL4 (Supplementary Materials Table S3). Moreover, a high fungal load and a small amount of plant pollen indicates that honeybees most likely gained sugar based diet. At the same time, PL4 honeybees had high amounts of *γ-*proteobacteria, *Orbales*, *Gilliamella* (35.32%, SD = 0.370) *α-proteobacteria Rhizobiales*, *Bartonella* (27.06%, SD = 0.560), known honeybee gut symbionts (Supplementary Materials Table S1A). *Gilliamella apicola* was found to be a dominant gut bacterium in honeybees and bumble bees, and this bacterium simultaneously utilizes glucose, fructose and mannose, and has the ability to break down other potentially toxic carbohydrates [50].

PL5 forager honeybees were collected in August and 27 taxa were identified in total from 16S amplicons analysed and 284 taxa in ITS2. PL5 diet was mainly based on *Helianthus* sp. (34.98%, SD = 1.616). PL5 had a higher number of *Lactobacillus* (44.31%, SD = 1.456) and *Gilliamella* (28.46%, SD = 0.129) species (Supplementary Materials Table S1A). The fungal load was medium (59.16%, SD = 2.225) with bioaerosol *Cladosporium* (11.23%, SD = 2.225) and *Mycosphaerella* (6.69%, SD = 1.358) as the main representatives (Supplementary Materials Table S1B). No pathogens were detected in PL5 (Supplementary Materials Table S3).

PL6 forager honeybees were collected in September, and 28 taxa were identified in total from 16S amplicons analysed and 191 taxa in ITS2. PL6 had the highest level of *Firmicutes* and *Lactobacillus* with 65.37%, SD = 0.731, and a slightly higher number of bacteria from the Comensalibacter group (Supplementary Materials Table S1A). PL6 honeybee diet was rich in *Calluna* pollen (88.26%, SD = 0.585), which displays low protein content and is considered to be a poor source of food for bees, and thus destructive for colony development [51,52]. The fungal load was moderate (11.26%, SD = 0.718) with *Penicillium* as a dominant genus with 0.96%, SD = 0.104 (Supplementary Materials Table S2). Pathogens belonging to *N. ceranae* and neogregarines were detected in PL6 (Supplementary Materials Table S3).

Generally, PCA analysis of stores from spring honeybees (PL1 and PL2), summer honeybees (PL3 and PL4) and autumn honeybees (PL5 and PL6) involved splitting data into five major components which accounted for 100% of the variation. It can be concluded from the PCA analysis that PL3 clearly differed from the others, and was mainly influenced by bacteria (b) and plants (p). PL2 and PL6 were similar to each other, bacteria (b) and plants (p) having the greatest impact as well. PL1 and PL5 were similar to each other. The greatest, albeit low, influence was caused by bacteria (b) and fungi (f). PL4, similarly to PL3, clearly stood out from the others, and was mainly influenced by plants (p) and fungi (f). It could also be generally seen that bacteria had the greatest influence on the variability of the plant pollen-bacteria-fungi system, followed by the plants, and then the fungi. The first two components accounted for over 53% of the variability of the entire system. Positive PC2 values may describe the summer months, and negative PC2 values the months closer to spring and autumn (Supplementary Materials Figure S1).

PC1 and PC3, the two main components, account for approximately 50% of the system variability (Supplementary Materials Figure S1a,b). It can be concluded that positive PC3 also described spring and autumn values, and negative PC3 values described the summer, which clearly differs from the others. Therefore, the third component described the season.

To summarise, we observed a prevalence of *Lactobacillus* and *Bartonella* species in honeybees collected during spring (April PL1, May PL2) and autumn months (September PL6), while in summer months, (June PL3, July PL4, August PL5) microbiome analyses showed the prevalence of *Gilliamella*, which is in agreement with previous findings [4,9,43]. In late July (PL4) the physiology of honeybees changes due to their adaptations to overwintering, and a role from two types of bacterial groups, i.e., *Lactobacillus* and *Giliamella* is suggested to be played in this process. However, increased *Lactobacillus* and *Giliamella* occurrence may simply be a consequence of a protein-rich diet. We observed fluctuations in the microbiome composition correlating with changes in the protein-richness of the pollen available in the environment. During spring and autumn, it is common for honeybees’ diet to be based on sugars ingested from honeydew. These high sugar diets can lead to fungal infections observed in April (PL1), May (PL2) and June (PL3) in honeybee samples. Probably, the detected pathogens were present in the honeybee colonies throughout the season but owing to the colony biology and well-balanced diet observed during May (PL4), June (PL3) and August (PL5), infections were less frequent among foragers and may have gone unnoticed in the whole colony screening tests.

### 2.2. Microbiome and Pollen Composition of Honeybees from UK, Greece, Spain, and Thailand

Forager honeybees from the UK were collected on the roof of the Fogg Building at Queen Mary University, London (UK1) and in the garden of the Natural History Museum, London (UK2), in July 2019. The mean day temperature in the first half of July was 21.93 °C (71.47 °F) with average humidity equal to 65% [53]. Their bacterial microbiota was mainly composed of *Firmicutes* (*Lactobacillus*), *γ-proteobacteria* (*Orbales*, *Gilliamella*), *α-proteobacteria* (*Rhizobiales*, *Bartonella*), *γ-*proteobacteria (*Neisseriaceae*, *Snodgrassella*), and *Actinobacteria* (*Bifidobacterium*) (Supplementary Materials Table S2A). In total, 42 and 93 taxa were identified (species level) from the 16S amplicon analysis, and 70 and 94 taxa (species level) in the ITS2 in the UK1 and UK2 groups, respectively. The UK1 sample contained modest amounts of fungi (12.59%) with the prevalence of plant pollen (87.41%) derived from Apiaceae (*Ammi*), Fabaceae (*Astragalus*), Resedaceae (*Reseda*), and Fabaceae (*Styphnolobium*). UK2 honeybees foraged on Hydrangeaceae (*Hydrangea*), and Bignoniaceae plants (Supplementary Materials Table S2B). The UK2 sample was dominated by fungi (90.80%), mainly from the Myxotrichaceae, which is reported to be a common hive fungus in Europe [54,55]. Moreover, the fungal pathogen *N. ceranae* was detected in the UK2 (Supplementary Materials Table S3).

Forager honeybees from Greece (GR1, GR2) were collected in November from two colonies inhabiting the garden of the Agricultural University of Athens. The mean day temperature in the first half of November was 18.53 °C (65.35 °F) with average humidity of 25% [56]. In total, 80 and 31 taxa were identified from the 16S amplicon analysis, and 42 and 96 taxa in the ITS2 in the GR1 and GR2 group respectively. Although the colonies were close, their microbiota and food preferences differed. GR1 microbiota were mainly Actinobacteria *(Bifidobacterium)*, Firmicutes *(Lactobacillus)*, and Cyanobacteria which reached 54.41%, 26.84%, and 11.66%, respectively. Cyanobacteria indicated some colony health problems most probably connected with the contamination of water used by the honeybee colony (Supplementary Materials Table S2A). Moreover, pathogens belonging to *N. ceranae* and neogregarines were detected in the GR1 (Supplementary Materials Table S3). The fungal load was miniscule (7.94%) and contained mainly fungi present in the air transferred as bioaerosols by wind, such as Mycosphaerella and *Cladosporium*. GR1 honeybees foraged mainly on Eudicotyledonae plants. GR2 microbiome contained α-proteobacteria (Rhizobiales, *Bartonella*), Firmicutes (*Lactobacillus*), and γ-proteobacteria (Orbales, *Gilliamella*) with 49.29%, 25.84%, and 9.78%, respectively. The amount of fungi was miniscule (6.03%) with the dominant taxon of Mycosphaerella reaching 1.09%. GR2 honeybees foraged mainly on Oleaceae (*Ligustrum*), Hydrangeaceae (*Hydrangea*), Myrtaceae (*Myrtus*), and Scrophulariaceae (*Buddleja*) (Supplementary Materials Table S2B).

Forager honeybees from Spain (ES1, ES2) were collected in November from experimental colonies located near Marchamalo. The mean day temperature in the first half of November was 18 °C (64.40 °F) with average humidity equal to 51% [57]. In total, 34 and 25 taxa were identified from the 16S amplicon analysis, and 31 and 13 taxa in the ITS2 in the ES1 and ES2 groups, respectively. ES1 contained γ-proteobacteria (Orbales, *Gilliamella*), and Firmicutes (*Lactobacillus*), at the level of 36.63%, and 33.11%, respectively (Supplementary Materials Table S2A). These honeybees most probably gained sugar based diet, since pollen DNA was hardly detected (Araliaceae (*Hedera*) 0.12% for ES1 and 0.04% for ES2). ES1 and ES2 had dominant fungal fraction containing spores transferred as bioaerosols by wind in the air, such as *Penicillium*, *Cladosporium* and *Mycosphaerella* (Supplementary Materials Table S2B). Additionally, pathogens belonging to *N. ceranae* and neogregarines were detected in ES2 (Supplementary Materials Table S3).

Forager honeybees from Thailand consisted of both: western honeybee (*Apis mellifera* TAI1 and TAI2) and Asian honeybee (*Apis cerana* TAI3 and TAI4). In Eastern Asia, these two bee genera inhabit the same locations, resulting in the transfer of pathogens from *Apis cerana* to *Apis mellifera*, as described for *Varroa destructor* and *Nosema ceranae*. Thai samples were collected in February, the best month in the year for honeybee colonies in Thailand. The mean day temperature in the first half of Februry was 33.33 °C (91.99 °F) with average humidity equal to 59% [58].

In western honeybee (*Apis mellifera* TAI1 and TAI2), in total 29 and 22 taxa were identified from the 16S amplicon analysis, and 50 and 94 taxa in the ITS2 in the TAI1, TAI2 groups, respectively. TAI1 contained high loads of Firmicutes (*Lactobacillus*) and *Bartonella* (Supplementary Materials Table S2A), and a high quantity of fungi such as *Aspergillus* (66.42%), Saccharomycetaceae (9.09%), and *Peniclilium* (6.53%) (Supplementary Materials Table S2B). A trace amount of plant pollen was detected in TAI1 (*Pterocarpus* with 0.16%, *Mimosa* 0.08%) indicating sugar based diet to have been the main source of forage (Supplementary Materials Table S2B). Bacterial microbiota of TAI2 was mainly composed of *Arsenophonus* bacteria (γ-proteobacteria, Enterobacteriales) 92.11% of which were insects’ intracellular symbionts. *Arsenophonus* species showed a broad spectrum of symbiotic relationships varying from parasitic son-killers to coevolving mutualists [59]. Moreover, pathogens belonging to *N. ceranae* and neogregarines were detected in TAI1 (Supplementary Materials Table S3). TAI2 honeybees foraged mainly on Asteraceae pollen (91.33%) and contained only a miniscule amount of fungi transferred as bioaerosols by wind in the air, as *Cladosporium* 4.32%.

From Asian honeybee (*Apis cerana*) in total 19 and 42 taxa were identified from the 16S amplicon analysis, and 59 and 85 taxa in the ITS2 in the TAI3 and TAI4 groups, respectively. *Apis cerana* samples contained 70.74% of *Lactobacillus* genus for TAI3, 27.17% for TAI4, *Gilliamella*, 33.45% for TAI4, and *Snodgrassella* with 3.60% and 13.13% for TAI3 and TAI4, respectively. Fungi present in the samples were related with the air bioaerosols, including *Aspergillus* 42.77% for TAI3, *Cladosporium* 59.03% and *Pleosporales* 30.38% for TAI4. The amount of plant pollen was miniscule (*Pterocarpus* with 0.42% for TAI3, *Mimosa* 0.15% for TAI4) indicating sugar based diet as the main forage for Thai *A. cerana* bees (Supplementary Materials Table S2). Moreover, pathogens belonging to *N. ceranae* and neogregarines were detected in TAI4 (Supplementary Materials Table S3).

**Figure 3:**
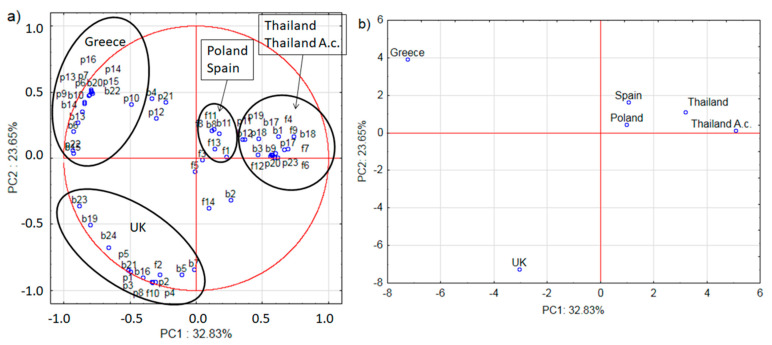
Loading plot (**a**) and score plot (**b**) of the principal components’ analysis (PC1 and PC2) carried out on the analytical data of the taxonomy detected in the world bees (Thailand A.c.—*A. cerana*). Small letters on loading plot (a): b—data obtained from bacteria NGS analysis, f—data obtained from fungal NGS analysis, p—data obtained from plant NGS analysis.

PCA analysis of the honeybees’ stores split data into five major components which accounted for 100% of the variation. PC1 and PC2 components accounted for nearly 57% of the variation, respectively 32.83% and 23.65 (Figure 3, figures a and b should be considered simultaneously).

The PCA analysis allowed us to determine the differences between bees from different countries. Four different areas were distinguished. Clear differences can be seen between bee samples from Greece, UK, Spain, Poland, and the two samples of bees from Thailand (Figure 3b).

PCA analysis shows Greek samples to markedly differ from the others, which is mainly influenced by bacteria (b) and plants (p). During the sample gathering, the weather conditions in Greece were most similar to those of Spain. Therefore, there must have been reasons for the Greek samples differences other than the weather. Most probably, nutrition was of the greatest importance in this case. The PCA analysis findings for UK bee samples also clearly differ from the others, mainly due to bacteria (b), then plants (p) and finally fungi (f). One can also distinguish samples from Spain and Poland from the other samples, which is mostly influenced by bacteria (b) and fungi (f), however, these are similar to the results for the samples from Thailand which are mostly influenced by bacteria (b), plants (p) and fungi (f). Bacteria, and, to a lesser extent, plants and fungi can be said to have the greatest influence on the variability of the system (Figure 3a,b).

Furthermore, ANOVA analysis confirms the correlation between the health status of honeybees and some of their bacterial microbiota (Supplementary Materials Table S4 and [60]). Bacterial groups such as Firmicutes (*Lactobacillus*); γ-proteobacteria, *Orbales*, *Gilliamella*, ***γ-***proteobacteria, Neisseriaceae, *Snodgrassella*, Enterobacteriaceae, and other unidentified bacteria had significantly different loads in healthy and in *Nosema ceranae* and neogreagarine infected honeybees. The load of fungi was always higher in infected honeybees (*p* = 0.002429) whereas the load of plant pollens was always higher in healthy honeybees (*p* = 0.030446).

Foragers are worker honeybees of a similar age, when they collect water, nectar, and pollen as well as supplements necessary for the colony to survive. All forager bees have similar function and physiological processes [61] and similar microbiota. Therefore, they should share similar microbiota. However, studies indicated that forager honeybees have a “contingent microbiome” dependent mainly on the food they forage [2,10,11]. This carries a danger, because with poor food resources, the microbiota will be inappropriate and non-functioning [62]. Currently, many factors influence bee microbiota e.g.,: monocultures, nutritional stress, pesticide exposure and agrochemicals, many of which exhibit antimicrobial properties, and thus contribute greatly to reductions in honeybee stress tolerance and disease resistance, leading to higher honeybee mortality, and a high rate of colony loss [5,63], pathogens which trigger bee malnutrition [64], changes in the composition of their microelements [65] and yeast content [19].

Intestinal pathogens such as microsporidia and neogragarines can strongly interfere with bee microbiota (Supplementary Materials Table S4). During *Nosema*-infection the honeybee intestine is covered by a layer of mature spores which is the cause of deprivation of the physiological function of the bee alimentary tract for food absorption [64]. Recent studies have revealed the *N. ceranae* infection course, showing a spring peak, and a subsequent decline in summer and autumn [19,66,67]. These studies also confirmed the seasonal pattern of *Nosema* infection, as in samples taken during April (PL1) and July (PL4, UK2) *N. ceranae* was detected. However, the presence of the parasite in Autumn samples (GR1, ES2) may indicate the colony health problem, and may pose a threat to overwintering. It is worth emphasizing that *N. ceranae* was found both in *Apis mellifera* (TAI1) and *A. ceranae* (TAI4) Thai samples. Besides *Nosema*-infection in samples PL4, PL6, GE1, ES2, TAI1, TAI4 also harboured other intestinal parasite such as neogregarines. Neogregarines, since their first detection in *A. mellifera* and *Bombus* sp. described in 1992 were linked to declines in bee populations [68,69,70]. Neogregarines, inhabit the intestines of many invertebrates and lead to the impoverishment of the host’s organism. Neogregarines’ interaction with their bee hosts had not been deciphered in full detail and still more studies need to be undertaken in regard to nutrient uptake and malnutrition, facilitating susceptibility to other diseases, etc.

Pollination is a crucial process for the maintenance of plant-based food supplies [71]. To maintain the health of honeybees, it is favourable to prevent the spread of disease, prevent exposure to insecticides and pesticides and provide a variety of plants to maintain optimal nutrition and microbiome throughout the season [72]. The use of NGS techniques in the identification of the pollen pool preferentially chosen by honeybees can provide strategies to maintain healthy colonies of bees. This technique can greatly expand and supplement knowledge based on long term observation for the most efficient “pollinator-friendly” plants [73,74,75]. This should help to create more effective pollinator beneficial plant species composition for “pollinator-friendly” gardens or to enrich the plant species on flower strips.

## 3. Conclusions

Honeybee dietary preferences, developed during the course of evolution, may not be currently favourable for honeybees. It is an urgent issue that should be carefully studied to aid bees to survive in the anthropogenic biosphere.

In our research, the composition of honeybee microbiomes was mainly determined by dietary preferences and forage availability. Even when colonies originated from one apiary, for instance from Spain or Greece, honeybees chose different plants to forage on. UK bees from a single highly urbanised area (London) exhibited particularly high diversity, chose different food sources and were otherwise prone to diseases. Furthermore, honeybees choosing a sugar based diet were more susceptible to pathogens (*Nosema ceranae* and neogregarines). The period when honeybees switch to the winter generation (longer-lived forager honeybees) was the time when the whole colony proved most sensitive to dietary perturbations.

Our findings are in line with limited other reports, that suggested honeybees from varying apiaries make independent decisions on the choice of pollen and nectar they forage upon [76]. As observed, colonies of bees located in close proximity may have different pollen composition in their gut. It remains unclear how bees decide which pollen to forage. However, flower structure, nectar volume, sugar content and composition were indicated to play a role in attracting bees [51,52,73]. Some studies indicated that honeybees primarily chose pollen rich in essential amino acids [51,52,73]. On the other hand, honeybees, because of their considerable energy need and having being weakened by disease, were unable to undertake forage flights to collect good quality pollen, and collected pollen of poorer nutritional quality. It is possible that evolutionary adaptation of bees has failed to benefit them in the modern anthropomorphised environment.

## 4. Highlights

1. The composition of honeybee microbiomes is mainly determined by their forage availability.
2. Honeybees were more susceptible to pathogens if they did not receive a well-balanced diet, and especially honeybees on sugar based diet were more prone to fungal pathogens (*Nosema ceranae*) and neogregarines. In most samples *Nosema* sp. and neogregarines parasitized the host bee at the same time.
3. The period when honeybees switch to the winter generation (longer-lived forager honeybees) is the most sensitive to diet perturbations, and hence pathogen attack, for the whole beekeeping season.

## 5. Materials and Methods

### 5.1. Honeybee Collection and DNA Isolation

Forager honeybees were recognized as bees returning to the hive and captured at the hive entrance about the midday. Forager honeybees from Poland were collected from one location in Lublin [51°15′ N 22°34′ E] each month from April to September 2018 (PL1-PL6). Forager honeybees from UK were collected from the roof of the Fogg Building of Queen Mary University, London (UK2) [51°52′ N 0°03′ W] and in the garden of the Natural History Museum, London (UK1) [51°29′ N 0°10′ W] in July 2019. Greek (GR1, GR2) samples of forager honeybees were collected in November 2017 from two colonies inhabiting the garden of The Agricultural University of Athens [37°59′ N 23°42′ E]. Forager honeybees from Spain (ES1, ES2) were collected in November 2017 from experimental colonies located at Marchamalo (Centro Apícola y Agroambiental de Marchamalo (CIAPA-IRIAF), Marchamalo, Spain [40°68′ N 3°21′ W]. Thai samples of forager bees consisting of both western honeybee (*Apis mellifera*) and Asian honeybee (*A. cerana*) were collected in February 2018 in the proximity of Chiang Mai University [18°50′ 98°58′ E], *A. mellifera* samples were marked TAI1 and TAI2, and *A. cerana* as TAI3 and TAI4. Genomic DNA was extracted from whole honeybees using QIAamp DNA Kit according to manufacturer’s instructions. Isolates were sent to the Biobank, Poland for NGS analysis.

### 5.2. NGS

NGS sequencing and the analysis of the 16S rRNA bacterial gene amplicon was based on the V3-V4 region and the ITS2 eukaryotic region for bee DNA samples. Amplicon libraries, were prepared using the *16S Metagenomic Sequencing Library Preparation*, *Preparing 16S Ribosomal RNA Gene Amplicons for the Illumina MiSeq System* (Illumina^®^ San Diego, CA, USA) protocol. Information about primers sequences, PCR conditions is shown in Supplementary Materials Table S5.

All data are available at https://www.ncbi.nlm.nih.gov/bioproject/PRJNA686953 (Submission Registration date: 21 December 2020).

### 5.3. Positive, Negative Control

The positive quality control for the V3-V4 region of the 16s rRNA gene was the DNA isolate derived from an ear swab. For the ITS2 region, it was DNA isolated from the *Saccharomyces cerevisiae* strain. PCR grade water was the negative quality control for both kinds of amplicons.

### 5.4. Purification, Clean-Up

The amplicons obtained were purified using magnetic beads (AMPure XP beads; Beckman Coulter Brea, CA, USA) according to Illumina^®^ protocol.

### 5.5. Library Pooling—Concentration, Normalization

Before pooling samples for libraries, the concentration was measured. The concentration [ng/uL] was measured using the NanoDrop™ 2000/c Spectrophotometer (Thermo Fisher Scientific Vienna; Austria) for each amplicon. Samples were diluted (PCR grade water) to the same concentration and pooled. To determine the final library concentration in [nM], the *NEBNext Ultra DNA Library Prep Kit for Illumina* (New England Biolabs^®^ Inc. Ipswich, MA; USA) protocol was followed. The final concentration of pooled libraries for sequencing was 8 pM.

### 5.6. Sequencing

Prepared libraries were sequenced on an Illumina MiSeq platform, 2 × 300 sequence reading in paired ends mode. The run contained PhiX libraries (PhiX Control Kit v3, Illumina^®^ San Diego, CA, USA), to serve as an internal positive quality control.

### 5.7. 16S rRNA Bacterial Gene Analyses

Reads from the sequencing run were imported into the QIIME 2 version 2019.10 artifact [77]. Then sequences were trimmed at first 21 bp for forward and reverse reads and truncated to 250 for forward reads and 240 for reverse reads. Reads were then denoised with DADA2 (Divisive Amplicon Denoising Algorithm v. 2) [78] and merged together. Sequences were aligned with MAFFT (Multiple Alignment using Fast Fourier Transform) [79] and used to construct a phylogeny with fasttree [80]. Rarefaction was performed with at least 14,096 sequences per sample for subsequent stages of the analysis. Taxonomic assignments of representative sequences were conducted using q2-feature-classifier with the sklearn classifier [81] trained on SILVA 132 database at 99% similarity level [82].

### 5.8. ITS2 Region Analyses

Analogous steps as for “*16S rRNA bacterial gene analyses*” were performed for the ITS2 analysis. Reads were trimmed and denoised separately, they were then merged for further analysis. Reads were then trimmed at first 21 bp for forward and reverse reads and truncated to 300 for forward reads and 215 for reverse reads. Taxonomic assignments were conducted analogous to 16S analysis with classifier trained on ITS gene clustered at 99% similarities within UNITE database released 04.02.2020 containing all eukaryotes [83]. Sampling depth was set to 34,100 sequences for the diversity analyses.

### 5.9. Analyses of Amplicon from Honeybee Samples

Amplicons for the 16S region and ITS2 were sequenced using the Illumina MiSeq platform. Data were trimmed and merged. For 16S analyses only full-length reads over 229 bp with medium length of all sequences at 414 bp were used. Sequences were assigned to taxonomy using classifier trained on SILVA 132 database with minimum similarity 90% of read matching to the reference. For ITS2 analyses only full-length reads over 269 bp with medium length of all sequences at 337 bp were used. Sequences were assigned to taxonomy using classifier trained on all eukaryotes UNITE database v8.2 with the minimum similarity of 90% of the read matching to the reference [84,85].

### 5.10. Screening for Pathogen Infected Honeybee Samples

Isolated DNA was used as the template for screening pathogens: *Nosema apis*, *Nosema ceranae*, *Nosema bombi*, tracheal mite (*Acarapis woodi*), any organism in the parasitic order Trypanosomatida, including Crithidia spp. (i.e., *Crithidia mellificae*), neogregarines including *Mattesia* and *Apicystis* spp. (i.e., *Apicistis bombi*), using PCR techniques described earlier [67,86,87,88]. Primers used for pathogen detection are listed in the Supplementary Materials Table S5. Detection of the pathogens in honeybee samples.

### 5.11. Statistical Analysis

Analyses of correlations and Principal Component Analysis (PCA) were performed using software Statistica (version 12.0, StatSoft Inc., Oklahoma; USA) at the significance level of α = 0.05. The analysis was used to determine the relationships between the bee sample and the bacterial group, plant group, and fungi group. The optimum number of principal components obtained in the PCA analysis was established based on Cattell’s criterion. The data matrix for the PCA of the Polish bees had 37 columns and 6 rows and of the world samples of bees had 61 columns and 6 rows (UK, Spain, Greek, Thailand and Poland) had 61 columns and 6 rows. The input matrix was auto-scaled. One-way ANOVA was performed to establish the correlation between honeybees’ health status and the bacteria, fungi and plant pollen detected. For the ANOVA test, the level of statistical significance was assumed to be α = 0.05, and the same level of statistical significance was used in all comparisons. The results for which *p* values are equal to, or less than, 0.05 were obtained differ significantly from each other.

## Data Availability

The data presented in this study are openly available in https://www.ncbi.nlm.nih.gov/bioproject/PRJNA686953 (accessed on 21 March 2021).

## Supplementary Materials

The following are available online at https://www.mdpi.com/2076-0817/10/3/381/s1, **Table S1.** Taxonomy analysis of 16S and ITS amplicon sequencing. **Table S1. A.** Taxonomy of 16S amplicon sequencing. Six sub-groups of three bees each were analysed. Table presents relative abundance of each of the listed bacterium (numbers as percentages) in relation to the entire amplicon. **Table S1. B.** Taxonomy of ITS2 amplicon sequencing. Six subgroups of three bees each were analysed. Hits for plants and fungi were grouped into separate fractions. % for either Plant or Fungi is a sum of all counts for each fraction (highlighted in grey). **Table S2. A.** Taxonomy of 16S amplicon sequencing. Four subgroups of two bees (UK, GR, ES) or four bees (TAI) each were analysed. Table presents relative abundance of each of the listed bacterium (numbers as percentages) in relation to the entire amplicon. **Table S2. B.** Taxonomy of ITS2 amplicon sequencing. Four subgroups of two bees (UK, GR, ES) or four bees (TAI) each were analysed. Hits for plants and fungi were grouped into separate fractions. % for either Plant or Fungi is a sum of all counts for each fraction (highlighted in grey). **Table S3.** Detection of the pathogens in honeybee samples. **Table S4.** The correlation between honeybees’ health status and the detected bacteria. Data with significant differences are written in bold. The ANOVA test, α = 0.05; p ≤ 0.05. **Figure S3.** Loading plot (a) and score plot (b) of the principal components analysis (PC1 and PC2) carried out on the analytical data of the taxonomy detected in all Polish bees (PL1 to PL6). **Table S5.** The list of primers and PCR conditions.

## Data Citation

Gancarz: M., Hurd, P.J., Latoch, P., Polaszek, A., Michalska-Madej, J., Grochowalski, Ł., Strapagiel, D., Gnat, S., Załuski, D., Rusinek, R., Starosta, A.L.., Krutmuang, P., Martín Hernández, R., Higes Pascual, M., Ptaszyńska, A.A. (2021). Dataset of the next-generation sequencing of variable 16S rRNA from bacteria and ITS2 regions from fungi and plants derived from honeybees kept under anthropogenic landscapes. Data in Brief.

## Lead Contact

Further information and requests for resources and reagents should be directed to and will be fulfilled by the Lead Contact, Aneta A. Ptaszyńska (aneta.ptaszynska@poczta.umcs.lublin.pl).
