# Supplementary material for: Amplicon Sequencing of Variable 16S rRNA from Bacteria and ITS2 Regions from Fungi and Plants, Reveals Honeybee Susceptibility to Diseases Results from Their Forage Availability under Anthropogenic Landscapes
Source: Pathogens. 2021 Mar 22;10(3):381. doi: 10.3390/pathogens10030381 (PMC8004708; doi:10.3390/pathogens10030381)
Supplement: Supplementary file 1 [file pathogens-10-00381-s001.pdf]

Supplementary materials

Scheme 1. Taxonomy analysis of 16S and ITS amplicon sequencing.

Table S1. A. Taxonomy of 16S amplicon sequencing. Six sub-groups of three bees each were analysed. Table presents relative abundance of each of the listed bacterium (numbers as percentages) in relation to the entire amplicon.

**Sample**<br>**Bacteria (%)**	**PL1 (1)**	**PL1 (2)**	**PL1 (3)**	**PL2 (1)**	**PL2 (2)**	**PL2 (3)**	**PL3 (1)**	**PL3 (2)**	**PL3 (3)**	**PL4 (1)**	**PL4 (2)**	**PL4 (3)**	**PL5 (1)**	**PL5 (2)**	**PL5 (3)**	**PL6 (1)**	**PL6 (2)**	**PL6 (3)**
Firmicutes Lactobacillus	21,20	21,43	21,39	44,22	41,81			9,48	10,61	19,91	18,45	19,60	42,83	44,36	45,74	66,04	64,59	65,48
γ-proteobacteria Orbales Gilliamella	6,99	6,95	7,33	26,97	27,15			55,68	52,38	34,91	35,42	35,63	28,51	28,31	28,55	6,21	5,54	5,02
α-proteobacteria Acetobacterales Commensalibacter	4,36	4,31	4,24	15,18	15,40			0,44	0,48	1,23	1,09	1,15	7,81	6,80	7,09	6,05	6,48	6,64
α-proteobacteria Rhizobiales Bartonella	59,70	59,62	59,40	5,19	6,16			2,40	2,87	27,36	27,40	26,41	12,04	12,03	11,14	12,43	12,82	12,49
γ-proteobacteria Neisseriaceae Snodgrassella	7,36	7,29	7,24	4,53	4,97	ND	ND	23,69	24,48	15,44	16,37	15,90	2,37	2,33	2,02	1,10	1,18	1,27
Actinobacteria Bifidobacterium	0,38	0,34	0,36	1,89	2,25			0,23	0,23	0,98	1,13	1,17	0,85	0,92	0,95	8,00	9,21	8,84
γ-proteobacteria Orbales Frischella	0,00	0,00	0,00	0,94	0,89			2,48	2,30	0,00	0,00	0,00	0,00	0,00	0,00	0,13	0,14	0,10
Bacteroidetes Dysgonomonas	0,00	0,00	0,00	0,59	0,81			0,00	0,00	0,00	0,00	0,00	0,00	0,00	0,00	0,00	0,00	0,00
Bacteroidetes Apibacter	0,00	0,00	0,00	0,45	0,55			5,36	6,51	0,00	0,01	0,01	5,05	4,87	4,08	0,00	0,00	0,00
γ-proteobacteria Enterobacteriales Serratia	0,00	0,00	0,00	0,00	0,00			0,00	0,00	0,09	0,04	0,03	0,38	0,25	0,28	0,00	0,00	0,00
Other	0,01	0,06	0,04	0,04	0,01			0,24	0,14	0,08	0,09	0,1	0,16	0,13	0,15	0,04	0,04	0,16

ND – no data.

Table S1. B. Taxonomy of ITS2 amplicon sequencing. Six subgroups of three bees each were analysed. Hits for plants and fungi were grouped into separate fractions. % for either Plant or Fungi is a sum of all counts for each fraction (highlighted in grey).

**Sample**	**PL1 (1)**	**PL1 (2)**	**PL1 (3)**	**PL2 (1)**	**PL2 (2)**	**PL2 (3)**	**PL3 (1)**	**PL3 (2)**	**PL3 (3)**	**PL4 (1)**	**PL4 (2)**	**PL4 (3)**	**PL5 (1)**	**PL 5(2)**	**PL5 (3)**	**PL6 (1)**	**PL6 (2)**	**PL6 (3)**
Plant (%)	1,32	2,34	1,76	93,49	85,25	92,46	89,38	90,19	86,67	12,06	13,8	10,53	42,08	37,91	41,77	87,91	89,41	88,78
Brassicaceae unassigned	0,01	0,01	0,01	66,23	60,61	57,97	16,13	14,76	11,07	0,19	0,15	0,07	0,00	0,00	0,00	0,01	0,06	0,06
Brassicaceae Raphanus	0,00	0,00	0,00	24,34	22,35	26,97	2,06	2,26	1,51	0,04	0,00	0,00	0,00	0,00	0,00	0,01	0,05	0,09
Brassicaceae Brassica	0,00	0,00	0,00	0,18	0,13	0,00	1,43	1,34	1,29	0,00	0,00	0,00	0,00	0,00	0,00	0,00	0,00	0,00
Brassicaceae Berteroa	0,00	0,00	0,00	0,01	0,04	0,00	0,13	0,04	0,07	1,28	2,02	1,86	0,08	0,04	0,10	0,00	0,00	0,00
Brassicaceae Erysimum	0,00	0,00	0,00	0,06	0,12	3,62	0,00	0,00	0,04	0,00	0,00	0,00	0,00	0,00	0,00	0,00	0,00	0,00
Asteraceae Scorzoneroides	0,00	0,00	0,01	0,74	0,53	3,21	61,40	61,42	61,08	0,00	0,00	0,00	0,00	0,00	0,00	0,00	0,00	0,00
Ericaceae Calluna	0,07	0,21	0,17	0,00	0,01	0,01	0,00	0,00	0,01	0,06	0,00	0,01	0,01	0,00	0,01	87,68	88,85	88,26
Asteraceae Helianthus	0,00	0,02	0,00	0,01	0,01	0,00	0,00	0,00	0,00	0,00	0,00	0,00	36,01	33,12	35,82	0,00	0,00	0,00
Polygonaceae Rumex	0,00	0,00	0,00	0,19	0,17	0,00	4,61	5,60	6,26	0,01	0,16	0,04	0,02	0,00	0,00	0,01	0,04	0,06
Fabaceae Ornithopus	0,13	0,46	0,21	0,11	0,12	0,00	0,10	0,22	0,16	4,65	5,87	3,80	1,50	1,22	0,95	0,10	0,12	0,05
Hypericaceae Hypericum	0,00	0,00	0,00	0,00	0,00	0,00	1,88	2,75	3,17	0,00	0,00	0,00	0,03	0,05	0,11	0,00	0,00	0,00

Plantaginaceae Plantago	0,00	0,00	0,00	0,21	0,10	0,00	0,00	0,01	0,01	2,08	2,29	2,38	0,00	0,00	0,00	0,00	0,00	0,00
other	1,11	1,64	1,36	1,41	1,06	0,68	1,64	1,79	2	3,75	3,31	2,37	4,43	3,48	4,78	0,1	0,29	0,26
Fungi (%)	94,14	93,19	94,16	6,5	14,72	7,54	10,62	9,8	13,33	87,61	85,5	88,82	57,61	61,93	57,95	12,01	10,58	11,18
Cladosporiaceae Cladosporium	2,20	2,50	4,55	2,37	4,44	0,01	1,31	1,12	1,19	11,88	9,80	11,06	9,74	13,79	10,17	0,20	0,30	0,43
Capnodiales unassigned	16,37	14,72	11,51	0,00	0,00	0,00	0,00	0,00	0,00	0,42	0,33	0,31	0,12	0,09	0,57	0,00	0,00	0,00
Pseudeurotiaceae Bettsia	4,78	4,50	3,33	0,01	0,10	0,00	0,14	0,27	0,19	9,80	10,90	12,90	0,28	0,10	0,18	0,10	0,05	0,04
Saccharomycetaceae Zygosaccharomyces	33,28	34,99	39,49	0,06	0,04	0,00	0,85	1,05	1,72	2,87	2,84	2,59	0,70	0,80	2,50	0,04	0,14	0,12
Aspergillaceae Aspergillus	2,30	2,59	1,29	0,23	0,66	0,00	2,65	2,62	6,01	16,55	15,60	15,79	0,24	0,84	0,38	0,02	0,11	0,00
Sclerotiniaceae unassigned	0,00	0,14	0,00	0,19	1,05	0,00	0,30	0,33	0,16	0,57	0,75	1,04	0,01	0,00	0,00	0,53	0,53	0,45
Mycosphaerellaceae Mycosphaerella	2,46	1,99	3,79	0,89	2,12	0,00	1,52	0,58	0,34	4,51	3,81	4,80	5,19	7,03	7,84	0,55	0,96	0,67
Pleosporaceae Alternaria	2,77	4,32	3,12	0,26	0,56	7,47	0,89	1,09	0,91	0,67	0,47	1,06	3,19	3,40	1,98	0,59	0,46	0,42
Aspergillaceae Penicillium	5,04	3,53	3,05	0,14	0,47	0,00	0,26	0,15	0,16	9,66	9,95	13,59	0,56	1,05	0,74	0,89	1,08	0,91
Myxotrichaceae unassigned	2,37	3,13	3,54	0,00	0,04	0,00	0,33	0,36	0,33	2,70	2,57	2,74	0,05	0,00	0,12	1,14	0,74	0,52
Sporidiobolaceae Rhodotorula	0,00	0,04	0,10	0,25	0,50	0,00	0,26	0,17	0,34	0,06	0,08	0,56	5,31	5,35	3,71	0,00	0,00	0,15
Leotiomycetes unassigned	0,01	0,00	0,03	0,03	0,06	0,00	0,00	0,08	0,03	0,21	0,26	0,27	3,00	4,25	3,38	0,09	0,03	0,08
other	22,56	20,74	20,36	2,07	4,68	0,06	2,11	1,98	1,95	27,71	28,14	22,11	29,22	25,23	26,38	7,86	6,18	7,39
Unassigned (%)	4,54	4,47	4,08	0,01	0,03	0,00	0,00	0,01	0,00	0,33	0,70	0,65	0,31	0,16	0,28	0,08	0,01	0,04

Table S2. A. Taxonomy of 16S amplicon sequencing. Four subgroups of two bees (UK, GR, ES) or four bees (TAI) each were analysed. Table presents relative abundance of each of the listed bacterium (numbers as percentages) in relation to the entire amplicon.

**Sample / Bacteria**	**UK1**	**UK2**	**GR1**	**GR2**	**ES1**	**ES2**	**TAI1**	**TAI2**	**TAI3**	**TAI4**
Country	UK	UK	Greece	Greece	Spain	Spain	Thailand	Thailand	Thailand	Thailand
Species	*A. mellifera*	*A. mellifera*	*A. mellifera*	*A. mellifera*	*A. mellifera*	*A. mellifera*	*A. mellifera*	*A. mellifera*	*A. cerana*	*A. cerana*
Firmicutes Lactobacillus	24,32	22,03	26,84	25,84	33,11	20,73	67,78	4,12	70,74	27,17
γ-proteobacteria, Orbales Gilliamella	21,24	18,46	0,49	9,78	36,63	0,70	2,02	1,16	9,91	33,45
α-proteobacteria,Acetobacterales Commensalibacter	0,94	0,55	0,00	0,50	0,35	0,38	6,73	0,04	0,00	1,51
α-proteobacteria, Rhizobiales Bartonella	18,51	0,00	0,14	49,29	4,53	66,67	13,30	0,86	0,00	9,88
γ-proteobacteria, Neisseriaceae Snodgrassella	20,19	27,84	0,05	4,30	0,95	4,41	2,78	0,73	3,60	13,13
Actinobacteria Bifidobacterium	7,22	17,54	54,41	5,55	8,05	6,21	4,71	0,27	0,47	2,63
γ-proteobacteria, Orbales Frischella	6,13	6,56	0,00	0,99	1,58	0,23	0,00	0,08	0,00	8,23
Bacteroidetes Dysgonomonas	0,00	0,00	0,00	0,00	5,99	0,00	0,00	0,00	0,00	0,00
Bacteroidetes Apibacter	0,00	0,00	0,02	0,00	0,13	0,00	0,00	0,00	3,67	0,00

γ-proteobacteria, Enterobacteriales Serratia	0,05	0,00	0,00	0,41	0,00	0,00	0,00	0,00	0,00	0,00
γ-proteobacteria Pseudomonas	0,00	0,47	0,19	0,00	8,40	0,03	0,02	0,43	0,00	0,94
γ-proteobacteria, Enterobacteriales Arsenophonus	0,00	0,00	0,00	0,00	0,00	0,00	0,00	92,11	0,00	2,42
γ-proteobacteria, Enterobacteriales Klebsiella	0,37	0,00	0,00	1,43	0,00	0,00	0,00	0,00	0,00	0,00
Cyanobacteria unassigned	0,28	1,78	11,66	0,01	0,01	0,00	0,00	0,13	0,00	0,15
γ-proteobacteria, Enterobacteri-aceae unassigned	0,19	0,23	0,32	0,48	0,04	0,01	0,04	0,00	0,00	0,04
Actinobacteria Cutibacterium	0,08	0,41	0,06	0,00	0,00	0,00	0,00	0,01	0,00	0,03
α-proteobacteria,Acetobacterales Bombella	0,03	0,01	0,00	0,03	0,04	0,43	0,10	0,01	7,05	0,04
Firmicutes, Lactobacillales Fructobacillus	0,01	0,64	0,00	0,51	0,00	0,01	2,11	0,00	4,49	0,00
Firmicutes, Lactobacillales Streptococcus	0,00	0,80	0,60	0,00	0,04	0,01	0,01	0,01	0,00	0,08
α-proteobacteria Rickettsiales	0,01	0,05	4,01	0,00	0,03	0,00	0,00	0,04	0,00	0,12
Fusobacteria Leptotrichia	0,01	0,25	0,05	0,00	0,01	0,00	0,00	0,00	0,00	0,00
γ-proteobacteria, Enterobacteriales Morganella	0,01	0,00	0,00	0,43	0,00	0,00	0,00	0,00	0,02	0,00
Firmicutes Veillonella	0,00	0,42	0,40	0,00	0,03	0,00	0,02	0,00	0,00	0,01
other bacteria	0,41	1,96	0,76	0,45	0,08	0,18	0,38	0,00	0,05	0,17

Table S2. B. Taxonomy of ITS2 amplicon sequencing. Four subgroups of two bees (UK, GR, ES) or four bees (TAI) each were analysed. Hits for plants and fungi were grouped into separate fractions. % for either Plant or Fungi is a sum of all counts for each fraction (highlighted in grey).

**Sample**	**UK1**	**UK2**	**GR1**	**GR2**	**ES1**	**ES2**	**TAI1**	**TAI2**	**TAI3**	**TAI4**
Country	UK	UK	Greece	Greece	Spain	Spain	Taiwan	Taiwan	Taiwan	Taiwan
Species	*A. mellif-era*	*A. mellif-era*	*A. mellif-era*	*A. mellif-era*	*A. mellif-era*	*A. mellif-era*	*A. mellif-era*	*A. mellif-era*	*A. cerana*	*A. cerana*
Fungi (%)	12,58	90,79	7,96	6,03	99,87	99,95	99,28	7,04	97,75	99,85
Saccharomycetaceae/ Lachancea	2,44	0,00	0,00	0,00	0,00	0,00	9,09	0,00	0,18	0,00
Aureobasidiaceae/ Aureobasidi-um	0,92	0,45	0,16	0,07	0,01	0,00	0,05	0,00	0,00	0,35
Pleosporaceae/ Alternaria	0,67	1,76	0,63	0,69	0,04	0,00	0,35	0,04	0,00	0,41
Cladosporiaceae / Cladosporium	1,36	1,40	1,00	0,48	0,18	0,00	0,88	4,32	0,00	59,03
Mycosphaerellaceae / Myco-sphaerella	1,36	5,20	1,24	1,09	0,02	0,00	0,15	0,06	0,11	0,08
Sordariaceae / unassigned	0,00	0,00	0,00	0,00	0,00	0,00	0,00	0,00	25,37	0,00
Botryosphaeriaceae / Lasiodiplo-dia	0,00	0,00	0,00	0,00	0,00	0,00	0,00	0,00	23,78	0,00
Aspergillaceae / Penicillium	0,58	0,25	0,59	0,00	98,22	0,00	6,53	0,00	0,01	0,00
Aspergillaceae / Aspergillus	0,11	0,00	0,00	0,00	0,04	0,02	66,42	0,03	42,77	1,94
Myxotrichaceae / unassigned	0,03	73,85	0,00	0,00	0,00	0,00	0,00	0,00	0,00	0,00
Saccharomycetales / Starmerella	0,01	0,02	0,01	0,00	0,01	99,81	0,02	0,01	0,09	0,00
Pleosporales	0,00	0,13	0,04	0,04	0,00	0,00	0,51	0,79	0,00	30,38
Apiosporaceae/ Arthrinium	0,00	0,00	0,00	0,00	0,00	0,00	4,02	0,00	0,01	0,00
other	5,10	7,73	4,29	3,66	1,35	0,12	11,26	1,79	5,43	7,66
Plants (%)	87,42	9,21	92,02	93,97	0,12	0,04	0,25	92,94	0,50	0,15

Apiaceae / Ammi	29,78	0,04	0,00	0,00	0,00	0,00	0,00	0,00	0,00	0,00
Fabaceae / Astragalus	23,55	0,00	0,00	0,00	0,00	0,00	0,00	0,00	0,00	0,00
Resedaceae / Reseda	14,43	0,00	0,00	0,00	0,00	0,00	0,00	0,00	0,00	0,00
Fabaceae / Styphnolobium	12,94	0,00	0,00	0,00	0,00	0,00	0,00	0,00	0,00	0,00
Hypericaceae / Hypericum	2,95	0,00	0,00	0,62	0,00	0,00	0,00	0,00	0,00	0,00
Oleaceae / Ligustrum	0,43	0,20	0,08	22,39	0,00	0,00	0,00	0,00	0,00	0,00
Hydrangeaceae / Hydrangea	0,31	1,22	0,51	38,51	0,00	0,00	0,00	0,00	0,00	0,00
Bignoniaceae / unassigned	0,00	5,58	0,00	0,00	0,00	0,00	0,00	0,00	0,00	0,00
Myrtaceae / Myrtus	0,00	0,85	0,00	7,96	0,00	0,00	0,00	0,00	0,00	0,00
Scrophulariaceae / Buddleja	0,00	0,12	0,12	8,07	0,00	0,00	0,00	0,00	0,00	0,00
Asteraceae / unassigned	0,00	0,09	0,00	0,00	0,00	0,00	0,00	91,33	0,00	0,00
Asteraceae / Hypochaeris	0,00	0,04	0,00	2,35	0,00	0,00	0,00	0,00	0,00	0,00
Eudicotyledonae / unassigned	0,00	0,00	89,14	0,00	0,00	0,00	0,00	0,00	0,00	0,00
Myrtaceae / Eucalyptus	0,00	0,00	1,49	0,44	0,00	0,00	0,00	0,00	0,00	0,00
Cucurbitaceae / Lagenaria	0,00	0,00	0,00	3,45	0,00	0,00	0,00	0,00	0,00	0,00
Asteraceae / Picris	0,00	0,00	0,00	2,31	0,00	0,00	0,00	0,00	0,00	0,00
Fabaceae / Pterocarpus	0,00	0,00	0,00	0,00	0,00	0,00	0,16	0,00	0,42	0,00
Fabaceae / Mimosa	0,00	0,00	0,00	0,00	0,00	0,00	0,08	0,67	0,00	0,15
Asteraceae / Bidens	0,00	0,00	0,00	0,00	0,00	0,00	0,00	0,65	0,00	0,00
Myrtaceae / Syzygium	0,00	0,00	0,00	0,00	0,00	0,00	0,00	0,00	0,06	0,00
Araliaceae / Hedera	0,00	0,00	0,07	0,00	0,12	0,04	0,00	0,00	0,00	0,00
other	3,03	1,07	0,61	7,87	0,00	0,00	0,01	0,29	0,02	0,00
Unassigned	0,00	0,00	0,02	0,00	0,01	0,01	0,47	0,02	1,75	0,00

Table S3. Detection of the pathogens in honeybee samples.

Pathogen name	Primers used for pathogen detection	reference	Presence in the studied sample
Nosema apis	APIS FOR 5′-GGGGGCATGTCTTTGACGTACTATGTA-3′ APIS REV 5′-GGGGGGCGTTTAAAATGTGAAACAACTATG-3′	Martín-Hernández et al. [66]	Not detected
Nosema ceranae	MITOC FOR 5′-CGGCGACGATGTGATATGAAAATATTAA-3′ MITOC REV 5′-CCCGGTCATTCTCAAACAAAAAACCG-3′	Martín-Hernández et al. [66]	Poland (PL1, PL4, PL6); United Kingdom (UK2); Greece (GR1); Spain (ES2); Thailand *Apis mellifera* (TAI1); Thailand *Apis cerana* (TAI4)
Nosema bombi 323bp	Nbombi-SSU-J F: CCATGCATGTTTTTGAAGATTATTAT R: CATATATTTTTAAAATATGAAACAATAA	Klee et al. [86]	Not detected
Tracheal mite (*Acarapis woodi*)	5′-TCTTCAATTTTAATTATACGT-3′ 5′-CAAAAATCAGAATAAATGTTGAAATA-3′	Yang et al. [87]	Not detected
Any organism in the parasitic order Trypanosomatida, including *Crithidia* spp. (i.e. *Crithidia mellificae*)	SEF: 5′- CTTTTGGTCGGTGGAGTGAT-3′ SER: 5′-GGACG TAATCGGCACAGTTT-3′	Meeus et al. [88]	Not detected
Neogregarines including *Mattesia* and *Apicystis* spp. (i.e. *Apicistis bombi*)	NeoF: 5′-CCAG CATGGAATAACATGTAAGG-3′ NeoR: 5′-GACAGCTTCC AATCTCTAGTCG-3′	Meeus et al. [88]	Poland (PL4, PL6); Greece (GR1); Spain (ES2); Thailand *Apis mellifera* (TAI1); Thailand *Apis cerana* (TAI4)

Table S4. The correlation between honeybees' health status and the detected bacteria. Data with significant differences are written in bold. The ANOVA test, $\alpha = 0.05$; $p \leq 0.05$.

	P value
Firmicutes ***Lactobacillus***	**0,001744**
γ-proteobacteria, Orbales ***Gilliamella***	**0,017399**
α-proteobacteria,Acetobacterales *Commensalibacter*	0,119138
α-proteobacteria, Rhizobiales *Bartonella*	0,068737
γ-proteobacteria, Neisseriaceae ***Snodgrassella***	**0,036455**
Actinobacteria *Bifidobacterium*	0,060827
γ-proteobacteria, Orbales *Frischella*	0,056530
Bacteroidetes *Dysgonomonas*	0,346594
Bacteroidetes *Apibacter*	0,324604
γ-proteobacteria, Enterobacteriales *Serratia*	0,283847
γ-proteobacteria *Pseudomonas*	0,244753
γ-proteobacteria, Enterobacteriales *Arsenophonus*	0,334947
γ-proteobacteria, Enterobacteriales *Klebsiella*	0,229814
Cyanobacteria *unassigned*	0,250109
γ-proteobacteria, Enterobacteriaceae ***unassigned***	**0,040270**
Actinobacteria *Cutibacterium*	0,177661
α-proteobacteria,Acetobacterales *Bombella*	0,303179
Firmicutes, Lactobacillales *Fructobacillus*	0,147061
Firmicutes, Lactobacillales *Streptococcus*	0,100507
α-proteobacteria *Rickettsiales*	0,314460
Fusobacteria *Leptotrichia*	0,223819
γ-proteobacteria, Enterobacteriales *Morganella*	0,308404
Firmicutes *Veillonella*	0,111057
**other bacteria**	**0,034245**

Supplementary materials Figures S1

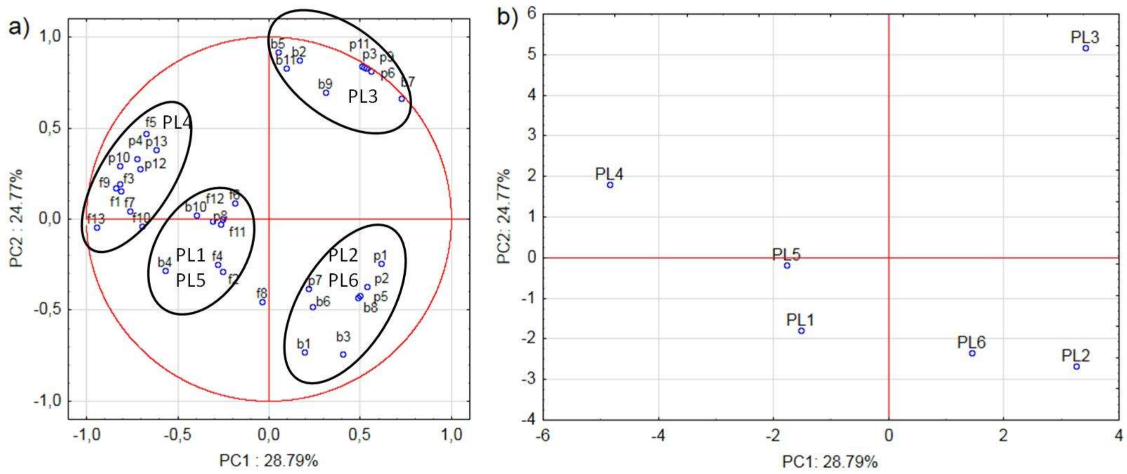

Figure S1. Loading plot (a) and score plot (b) of the principal components analysis (PC1 and PC2) carried out on the analytical data of the taxonomy detected in all Polish bees (PL1 to PL6).

Table S5. The list of primers and PCR conditions.

Gene	Region	Name	Sequences (5′-3′)	AT (°C)	Product size (bp)	References
**16S rRNA**	V3-V4	341f	CCTACGGGNGGCWGCAG	55	444	Klindworth et al., 2013 [83]
		785r	GACTACHVGGGTATCTAATCC			
**5.8S rRNA**	ITS2	ITS3	GCATCGATGAAGAACGCAG	60	330	White et al., 1990 [84]
		ITS4	TCCTCCGCTTATTGATATGC			
